# Supplementary material for: A Single Amino Acid Substitution in Porcine Reproductive and Respiratory Syndrome Virus Glycoprotein 2 Significantly Impairs Its Infectivity in Macrophages
Source: Viruses. 2022 Dec 18;14(12):2822. doi: 10.3390/v14122822 (PMC9781675; doi:10.3390/v14122822)
Supplement: Supplementary file 1 [file viruses-14-02822-s001.zip › viruses-2070947-supplementary.pdf]

Figure S1

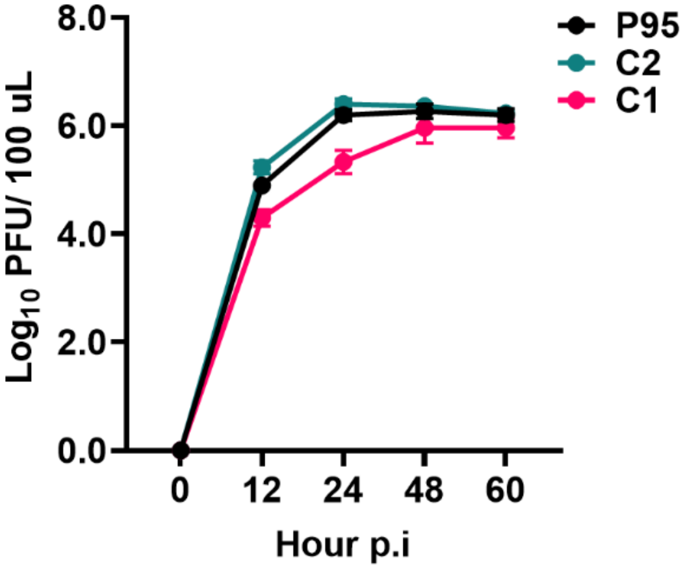

Figure S1. Multistep growth curve of NCV1 P95 and its derived plaque-clones in MARC-145 cells. MARC-145 cells in 24-well plates were inoculated with indicated viruses at a MOI of 0.1. Samples of culture supernatants were collected at indicated time points post-infection and virus titers were determined by a plaque assay in MARC-145 cells. Data are expressed as mean ± SEM of the three independent experiments.

Figure S2

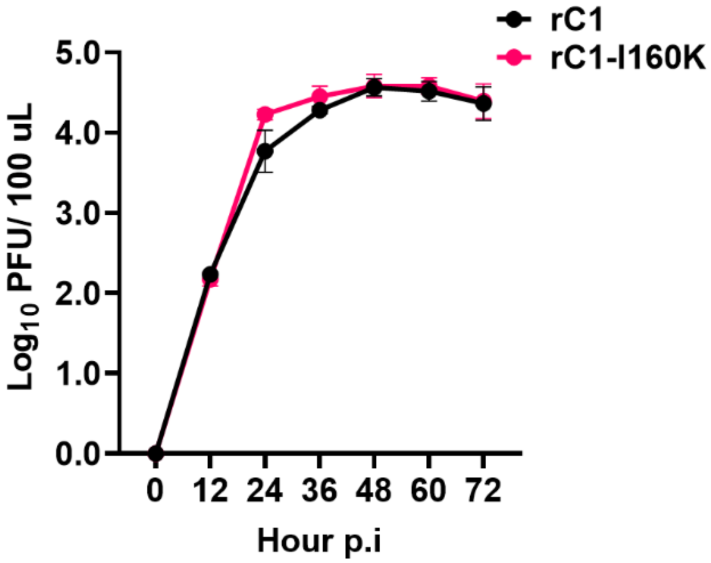

Figure S2. Multistep growth curve of rNCV1 and rNCV1-I160K mutant. MARC-145 cells in 24-well plates were inoculated with indicated viruses at a MOI of 0.1. Samples of culture supernatants were collected at indicated time points post-infection and virus titers were determined by a plaque assay in MARC-145 cells. Data are expressed as mean ± SEM of the three independent experiments.

Figure S3

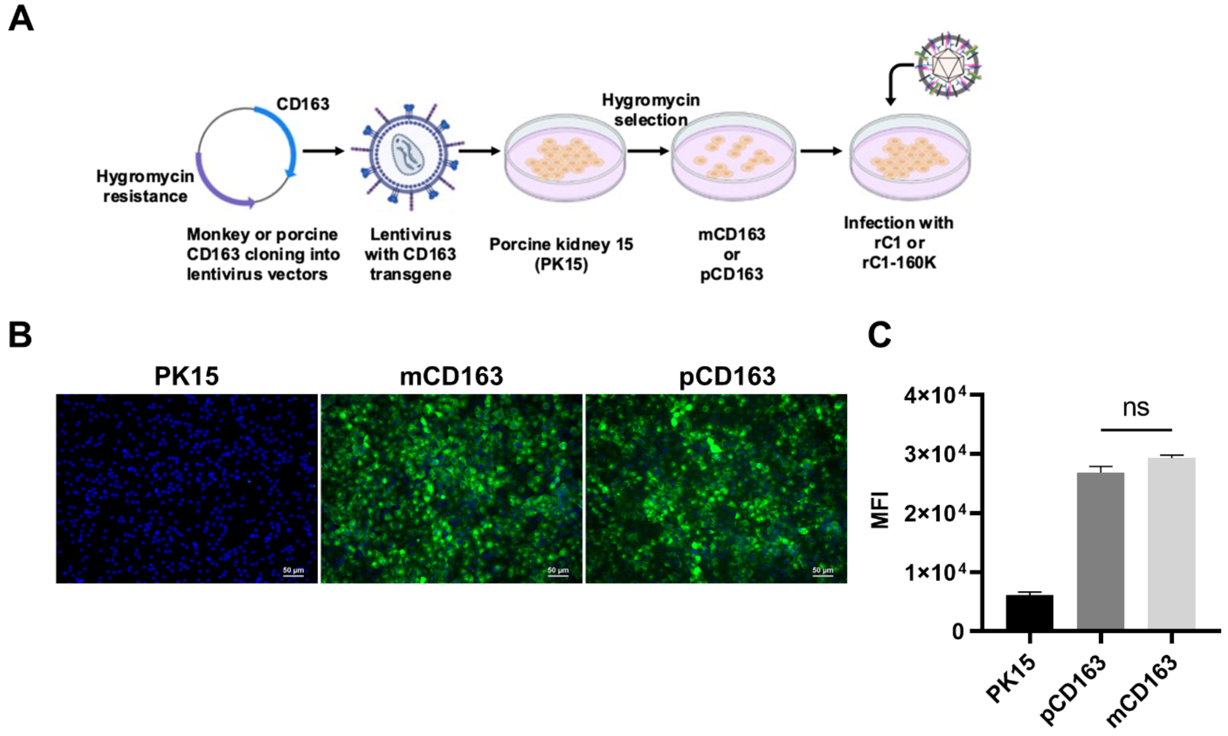

Figure S3. Generation of PK15 cell line expressing pCD163 or mCD163. (A) CD163 sequence of both species were cloned independently into the lentivirus vector. Recombinant Lentiviruses carrying either pCD163 or mCD163 were harvested from the culture supernatant of HEK-293T cells transfected with lentivirus vector plasmid and packaging plasmid. PK15 cells were transduced with the recombinant lentiviruses and non-transduced cells were killed with hygromycin B selection antibiotic (100ug/ml). Four round of cell passage was performed under the hygromycin B selection pressure before the cell uses in infection studies. (B) Immunofluorescence visualization of a CD163 protein expression. The pCD163 and mCD163 was detected with an anti-CD163 primary antibody. DAPI was used to stain the nuclei. Scale bar = 50. (C) Mean fluorescence intensity (MFI) of pCD163 or mCD163 expression on transduced PK15 cells.

Figure S4

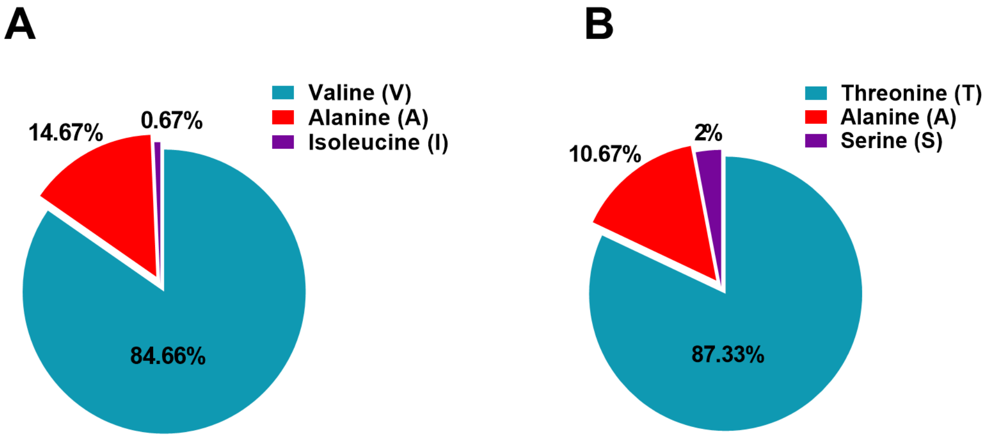

Figure S4. Frequency of unwanted mutations in rNCV13-K160I virus detected in natural PRRSV strains**.** The open reading frame 1ab amino acid sequence of rNCV13-K160I was BLAST and top 150 sequences were aligned using multiple sequence alignment tool. Shown are pie-charts showing the percentage of amino acid residues found at position 462 (A) and 718 (B) in poly protein 1ab protein.

Figure S5

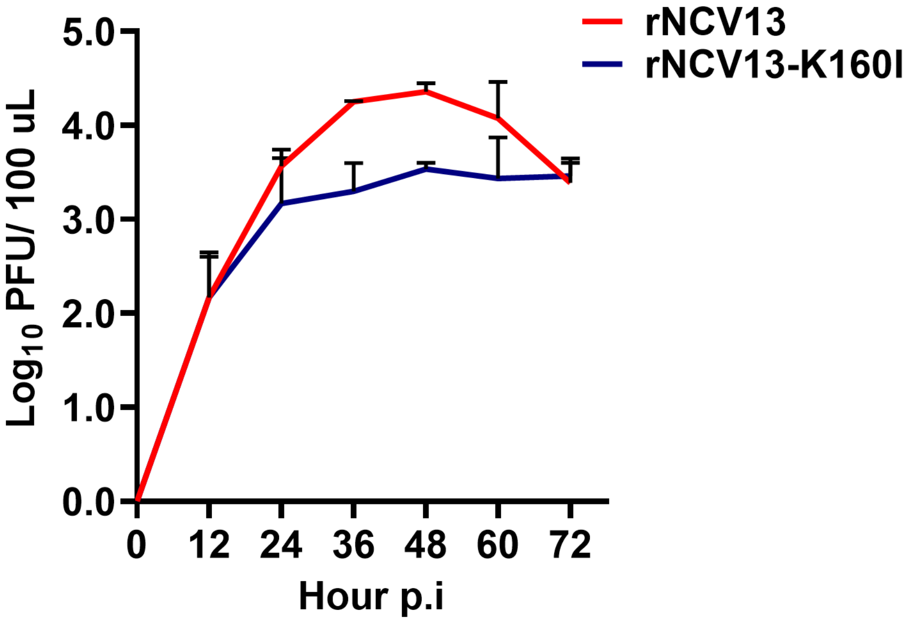

Figure S5. Multistep growth curve of rNCV13 and rNCV13-K160I mutant. MARC-145 cells in 24-well plates were inoculated with indicated viruses at a MOI of 0.1 per cell. Samples of culture supernatants were collected at indicated time points post-infection and virus titers were determined by a plaque assay in MARC-145 cells. Data are expressed as mean ± SEM of the three independent experiments.

Table S1. Mutations common in the P95, C1, and C2 viruses

Nucleotide position [a]	Nucleotide change	Protein affected	Amino acid position [b]	Amino acid change
1830	C→T	nsp2	610	Silent
1848	G→A	nsp2	616	Silent
2045	T→C	nsp2	682	V→A
2444	C→T	nsp2	815	T→I
2565	A→G	nsp2	855	Silent
2618	T→C	nsp2	873	V→A
2915	C→T	nsp2	972	T→I
3201	C→T	nsp2	1067	Silent
3696	C→T	nsp2	1232	Silent
3872	A→G	nsp2	1291	Silent
4752	T→C	nsp3	1584	Silent
4831	T→C	nsp3	1611	I→T
6102	C→T	nsp5	2034	Silent
6103	T→C	nsp5	2035	Silent
6116	A→G	nsp5	2039	Silent
6182	A→G	nsp6	2061	Silent
6185	C→T	nsp6	2062	Silent
6191	G→C	nsp6	2064	Silent
6227	G→C	nsp7	2076	M→I
6235	A→G	nsp7	2079	N→D
6514	A→G	nsp7	2172	S→G
6627	T→C	nsp7	2209	Silent
7277	A→G	nsp9	2426	Silent
7520	A→G	nsp9	2507	Silent
7775	C→T	nsp9	2592	Silent
8770	G→A	nsp9	2924	R→K
9393	T→C	nsp10	3132	Y→H

9482	T→C	nsp10	3161	Silent
9502	A→G	nsp10	3168	Silent
9557	C→T	nsp10	3219	Silent
9692	C→T	nsp10	3231	Silent
10028	T→C	nsp10	3343	Silent
10205	T→C	nsp10	3402	Silent
10535	A→G	nsp11	3512	Silent
10661	A→G	nsp11	3554	Silent
11099	A→G	nsp12	3700	Silent
11206	A→G	nsp12	3736	K→R
11425	C→T	nsp12	3809	A→V
11460	G→A	nsp12	3854	V→I
11463	A→G	nsp12	3822	I→V
11519	G→T	GP2	10	L→F
11781	A→C	GP2	98	I→L
11853	T→G	GP2	121	H→Q
11859	A→G	GP2	123	Silent
11861	G→A	GP2	124	Silent
12024	T→C	GP2	178	Silent
12425	G→A	GP3	105	A→T
13202	A→G	GP4-GP5 intergenic region		Silent
13298	A→G	GP5	32	N→D
13461	C→T	GP5	86	H→Y
13694	G→A	GP5	164	G→R
14576	C→T	N	90	Silent

[a]Nucleotide positions are based on PRRSV NCV-1 genome (GeneBank accession number ON950548)

[b]For nonstructural proteins (nsp2-nsp12), amino acid positions refer to the polyprotein pp1ab sequence of PRRSV NCV-1.
